# Effect of Zr Doping on the Magnetic and Phase Transition Properties of VO_2_ Powder

**DOI:** 10.3390/nano9010113

**Published:** 2019-01-18

**Authors:** Jing Xu, Haiying Wang, Zhihong Lu, Zhenhua Zhang, Zhaorui Zou, Ziyang Yu, Ming Cheng, Yong Liu, Rui Xiong

**Affiliations:** 1 School of Physics and Technology and the Key Laboratory of Artificial Micro/Nano Structures of Ministry of Education, Wuhan University, Wuhan 430072, China; xujinghenan@126.com (J.X.); wanghaiy@whu.edu.cn (H.W.); zzhua_mag@whu.edu.cn (Z.Z.); zrzou@whu.edu.cn (Z.Z.); tommyu91@163.com (Z.Y.); chengming-phy@outlook.com (M.C.); 2 College of Physics and Materials Science, Henan Normal University, Xinxiang 453007, China; 3 School of Materials and Metallurgy, Wuhan University of Science and Technology, Wuhan 430072, China; zludavid@live.com

**Keywords:** V-V dimer, magnetic properties, phase transition temperature, Zr-doped

## Abstract

In this work, V_1−x_Zr_x_O_2_ powder (x = 0, 0.01, 0.02, 0.04) was synthesized by two step hydrothermal method. The micro-topography, magnetic and phase transition properties have been investigated using various measurement techniques. All prepared V_1−x_Zr_x_O_2_ powder samples exhibit monoclinic structure at room temperature. With the Zr^4+^ ions doping concentration increased, the shapes of VO_2_ particles change from spherical to rectangular slice. Besides, the saturation magnetic moment of the samples decrease with the increase of doped Zr^4+^ ions concentration, while their phase transition temperature increase gradually with Zr ions doping at a rate of around 2 °C/at% on average. We investigated the Zr doping effects on V-V dimers and confirmed the role of V-V dimers in phase transition. We speculate that more V-V dimers form with Zr doping by magnetic measurements, which result in the monoclinic phase of Zr-doped VO_2_ sample is more stable than rutile phase. Therefore the phase transition temperature is elevated by Zr doping in our experiment. We further consider that the VO_2_ phase transition should be ascribed to Peierls transition caused by the changing of V-V dimers.

## 1. Introduction

Vanadium dioxide (VO_2_) is a first-order phase transition material, that transforms itself from a low temperature monoclinic phase [P21/c] to a high temperature rutile phase [P42/mnm] at 68 °C, which resulting in significant changes to its resistance and optical properties [1,2,3]. Changes to its metal-insulating transition properties can also be triggered by the application of an external electric field or stress [4,5,6]. Therefore, the unique phase transition properties of VO_2_ derived materials have been exploited for applications towards smart windows [7], thermoelectric materials [8,9], resistance switch [10,11,12] and temperature measurement devices [13,14].

However, there are a few problems still limit its practical application, for example, a high phase transition temperature (Tt) of VO_2_ [15,16,17]. Ion doping process have been developed to try and address this issue. Doping mainly divided into two types: The first type involves the use of high valence state cations such as W^6+^, Mo^5+^, Nb^5+^, which are known to decrease the phase transition temperature by introducing extra electrons into the VO_2_ sample [18,19,20]; The second type is to utilizes low valence state cations, such as Al^3+^, Cr^3+^, to increase the phase transition temperature of the VO_2_ derived materials [21,22,23]. However, there is no clear explanation currently existing to clarify the beneficial effects of substitution V^4+^ ions with Ti^4+^ ions or Zr^4+^ ions that have the same valence state. Zhang et al. reported that the phase transition temperature (Tt) rose from 42 °C to 56.7 °C in cooling process; while Tt decreased at Zr/V ratio from 0 to 8.5% and then increased with increase of Zr/V ratio in heating process [24]. Shen et al. reported that Zr doping could decrease the phase transition temperature and improve solar regulation rate simultaneously, the phase transition temperature decreased to 64.3 °C when the zirconium doping concentration was up to 9.8% [25]. Li et al. synthesized VO_2_ films with a sol-gel method and found that the effect of Zr^4+^ doping decreased phase transition temperature by approximately 1 °C/at% on average [26]. Lu et al. reported that the phase transition temperature (Tt) decreased with the increase of zirconium doping concentration, the Tt reduced to 50 °C when the zirconium doping concentration up to 2 wt% [27]. Therefore, it is necessary to study the influence of 4-valent ion dopants (Zr^4+^ ions) on the phase transition temperature of VO_2_ samples.

In this work, un-doped, 1%, 2% and 4% Zr-doped VO_2_ powders were fabricated using a hydrothermal method and their phase transition temperatures, micro-topography and magnetic properties were investigated using different measurement techniques. Experimental results revealed that the phase temperature of these VO_2_ samples increased as the amount of Zr doping increased, which may due to Zr mediate the transformation of free V^4+^ ions into V-V dimers that are formed as zigzag chains at low temperature.

## 2. Experimental Methods

Analytical grade reagents were used for the synthesis without further purification. Highly crystalline V_1−x_Zr_x_O_2_ (x = 0, 0.01, 0.02, 0.04) samples were synthesized via a two-step hydrothermal method through the reaction of vanadyl acetylacetonate and ethylene glycol, with zirconium nitrate pentahydrate used as a doping source. 0.4 g of vanadyl acetylacetonate and a specified molar ratio of zirconium nitrate pentahydrate were dissolved in an aqueous solution of glycol. This mixed solution was then heated in a 100 mL autoclave at 200 °C for 24 h to obtain a powder which was washed several times and then dried at 60 °C in a drying oven. The resultant powder was then annealed under a high-purity Ar atmosphere at 500 °C for 15 h to obtain highly crystalline un-doped VO_2_ powder, 1% Zr-doped VO_2_ powder, 2% Zr-doped VO_2_ powder and 4% Zr-doped VO_2_ powder.

The structure of the VO_2_ powder samples were characterized by X-ray diffraction (XRD, D8 Brucker, Karlsruhe, Germany). The surface micro-topography of the samples were observed using field emission scanning electron microscope (FE-SEM, Zeiss SIGMA, Carl Zeiss Microscopy Ltd., Cambridge CB1 3JS, UK) and their phase transition temperatures obtained from their thermal hysteresis loops. The elemental compositions of the samples were determined by X-ray photoelectron spectroscopy (XPS, ESCALAB 250Xi, Thermo Fisher Scientific, Waltham, MA, USA) and the magnetic properties were analyzed using a vibrating sample magnetometer (VSM, Quantum Design PPMS, San Diego, CA, USA) using a magnetic field of 10 K Oe.

## 3. Results and Discussion

**Figure 1:**
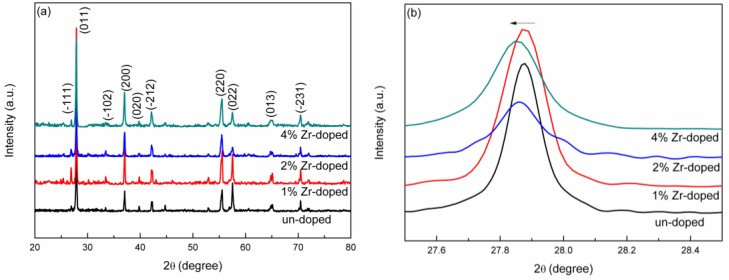
(**a**) X-ray diffraction (XRD) patterns and (**b**) Enlarged X-axis patterns from 27.5° to 28.5° of un-doped, 1% Zr-doped, 2% Zr-doped and 4% Zr-doped VO_2_ samples.

The crystal phase structures of samples un-doped, 1% Zr-doped, 2% Zr-doped VO_2_ and 4% Zr-doped VO_2_ powder were determined from their XRD patterns measured for a 2θ range from 20° to 80° shown in Figure 1a, which revealed that all the samples have monoclinic structure (space group P21/c, standard lattice parameters form PDF card: a = 0.575 nm, b = 0.454 nm, c = 0.538 nm and β = 122.64°, JCPDS No. 43-1051). No oxides of Zr were detected in the diffraction patterns, even when the Zr content was as high as 4%, which indicates that the Zr had been effectively doped into the VO_2_ lattice. Figure 1b shows a magnified view of the XRD peak corresponding to the (011) lattice plane. The peak shifted slightly towards the small angle, indicating that Zr doping had resulted in an expanded lattice [26,27]. The reason is that the doped Zr^4+^ ions have a higher radius (0.072 nm) than the V^4+^ ions (0.058 nm). The grain size of the samples decreased gradually with the increased of Zr doping concentration, which indicates that Zr doping increases the number of defect sites to promote the formation of multiple nucleation centers [28].

The interplanar spacing values were calculated using the formula 2dsin θ = γ, where θ is the Bragg’s diffraction angle and γ is the wavelength of the X-ray which equals to 0.154 nm (Cu Kα radiation). The grain sizes of the selected lattice plane were calculated using the Debye-Scherrer’s equation: D = Kγ/Bcos θ, where K is the Scherrer’s constant which equals to 0.89, γ is the wavelength of X-ray, B is the full width at half-maximum (FWHM) (in rad) and θ is the Bragg’s diffraction angle (in degrees). The unit cell volume for monoclinic VO_2_ was calculated using V = a*b*c*sin β, where a, b and c are lattice constants and β is the angle of b-axis with the AOC plane. The lattice parameters and the volume of the unit cells of un-doped, 1% Zr-doped, 2% Zr-doped and 4% Zr-doped VO_2_ samples are showed in Table 1. The calculated results of the lattice volumes increased gradually with the increase of Zr doping concentration, because the doped Zr^4+^ ions have a larger ionic radius than that of V^4+^ ions.

**Figure 2:**
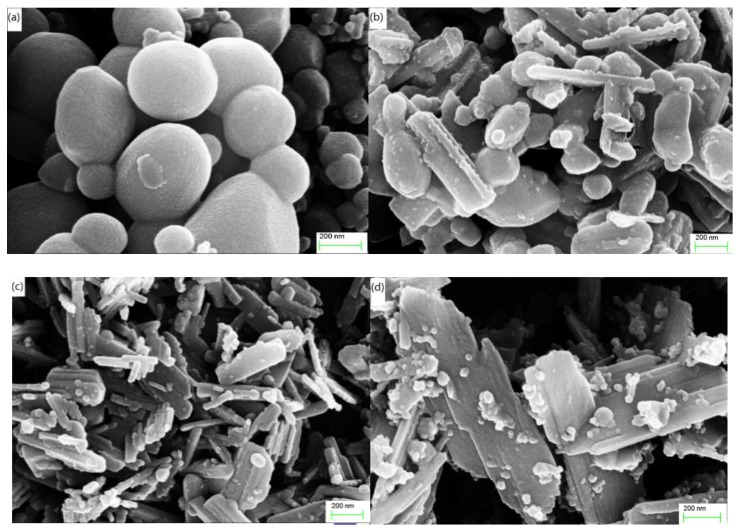
(**a**–**d**) FE-SEM (field emission scanning electron microscope) micro-topographic images of the un-doped, 1% Zr-doped, 2% Zr-doped and 4% Zr-doped VO_2_ samples, respectively. All images were observed in InLens mode with an accelerated voltage of 15 kV.

The effect of Zr doping on the morphology of VO_2_ samples was measured using FE-SEM analysis (Figure 2a–d). The un-doped VO_2_ samples are nano-sphere with some of them aggregate together (Figure 2a), while Zr doped VO_2_ samples exhibit both spherical and rod-like particles (Figure 2b,c). With the concentration of Zr dopant increased, some of the rod-like particles were found to combine to form extended slices. For example, the SEM images of 4% Zr-doped VO_2_ samples shown in Figure 2d reveal that the majority of VO_2_ particles combined into a sheet-like form, with only a few remaining in their granular ball shape. Due to the large differences of electron-negativity between V (1.63) element and Zr (1.33) element, the bond energy of Zr-O bond is larger than that of V-O band. The V-O bond was replaced by Zr-O band and the Gibbs free energy per unit volume of the system goes down with Zr^4+^ ions doping into VO_2_ lattice. And the critical nucleation energy decreases, simultaneously, which leads to the higher density of crystal nucleus. In a word, the doping of Zr ions increase the number of defect-nucleation sites [28] and serve to prevent the growth of VO_2_ in specific directions. The grain size of un-doped, 1% Zr-doped, 2% Zr-doped and 4% Zr-doped VO_2_ samples decreasing gradually, while grain density increase gradually with the increase of Zr doping concentration.

**Figure 3:**
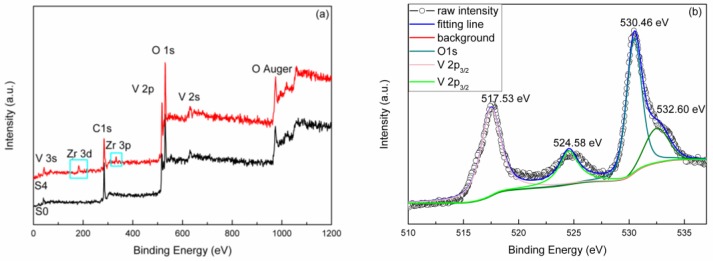

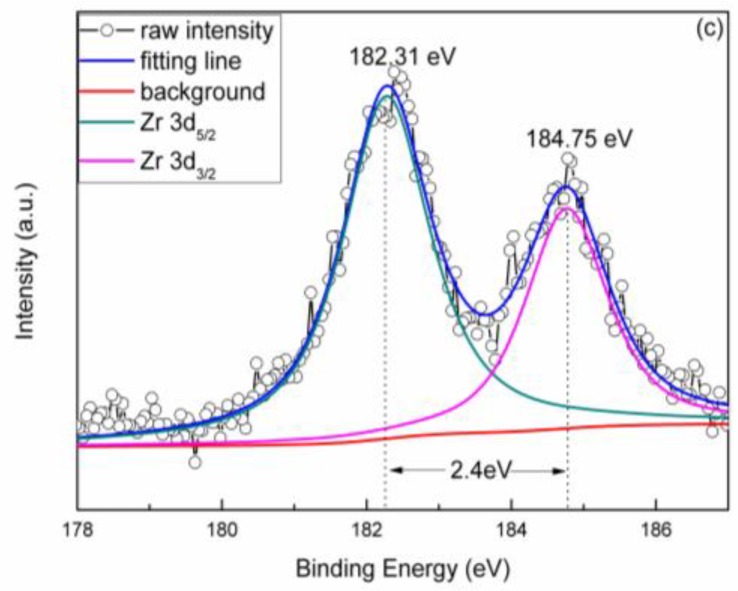
(**a**) XPS (X-ray photoelectron spectroscopy) spectra of un-doped and 4% Zr-doped VO_2_ samples with binding energy from 0 to 1200 eV; (**b**) Core level for V 2p and O 1s of 4% Zr-doped VO_2_ samples; (**c**) Core level for Zr 3d of 4% Zr-doped VO_2_ samples.

The element chemical states of the samples were investigated using XPS analysis, with XPS spectra for Zr, V and O element. Figure 3a shows the full range spectra of the un-doped and 4% Zr-doped samples, binding energy (BE) ranging from 0 to 1200 eV. Two weak peaks appeared at BE around 190 eV and 320 eV were marked with cyan square in the 4% Zr-doped samples corresponding to Zr 3d and 3p energy levels [29], respectively. This demonstrates the Zr ions successfully doped into the VO_2_ lattice. Due to the BE of V 2p and O 1s is approaching, their core level spectra are shown together in Figure 3b. The peaks at BE of 517.53 eV and 524.74 eV correspond to V^4+^ valences, V 2p_3/2_ and 2p_1/2_ states, respectively [18]. And the distance between doublets was 7.2 eV consistent with Zou et al. report [18]. A weak O 1s peak at 520.96 eV corresponding to O 1s X-ray satellite peak was also present [30]. A main peak appeared at 530.59 eV corresponding to a typical lattice O^2−^ absorption that has been reported for many metal oxides (e.g. TiO_2_ and VO_2_) [31,32]. A peak appeared with a BE of 532.56 eV was assigned to C=O and hydroxyl groups that probably result from contamination of sample [31]. In Figure 3c, two peaks appeared at BE of 182.31 eV and 184.75 eV which correspond to Zr 2p_3/2_ and 2p_1/2_ energy levels, respectively [33] Thus confirming that Zr^4+^ had been effectively doped into the VO_2_ lattice.

**Figure 4:**
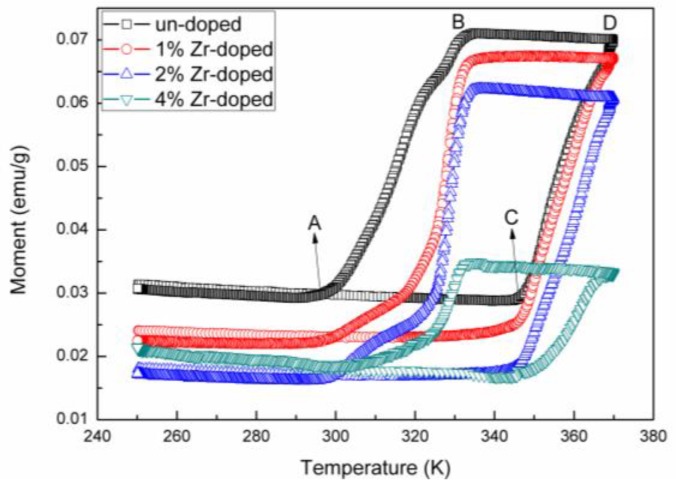
Moment vs. Temperature curves of un-doped, 1% Zr-doped, 2% Zr-doped and 4% Zr-doped VO_2_ samples from 250 K to 370 K in a 10 k Oe magnetic field.

The magnetic moment versus temperature curves of the samples reveal abrupt increases in their magnetic moments for increasing temperature in Figure 4. (Moment vs applied field curves of un-doped VO_2_ powder samples before and after phase transition (300K and 350K are showed in Figure S1). The rutile phase of un-doped VO_2_ samples have the largest magnetic moment of 0.07 emu/g, while the 4% Zr-doped samples have the lowest magnetic moment of 0.039 emu/g. For VO_2_ rutile phase, it is known that V^4+^ ions are periodically arranged in straight chains, with every V^4+^ ion having a free electron that contributes a magnetic moment of s = 1/2 [34]. The magnetic moment of the rutile phase arises from the presence of free V^4+^ ions and the doped Zr^4+^ ions contribute approximately zero to the overall magnetic moment. Therefore, the doping of Zr^4+^ ions leads to an overall decrease in the saturation magnetic moment of the VO_2_ derived material.

For VO_2_ monoclinic phase, the main difference with the VO_2_ rutile phase is the formation of V-V dimers [35]. The paired V-V dimers that are formed as zigzag chains arranged in an approaching antiparallel direction, approximately contributing a zero magnetic moment. Besides the paired V-V dimers, some unpaired free V^4+^ ions may also be present in the monoclinic [36]. For Zr doped VO_2_ samples, the total magnetic moment is given by M_total_ = M_dimers_ + M_free_ + M_Zr_. Therefore, when Zr is doped into the VO_2_ lattice, the magnetic moment decreased significantly, which means that it not only related to the substitution of V^4+^ ions but also the formation of V-V dimers. It is proposed that Zr^4+^ act as initiators to promote conversion of free V^4+^ ions into zigzag V-V dimer that results in an overall decrease in the total magnetic moment. The mechanism of Zr^4+^ ion dopants which promotes dimer formation is unknown, however it is clear that free V^4+^ ions in the high temperature state have a much larger magnetic moment than V-V dimers in the low temperature state.

The phase transition temperatures of the doped materials were obtained from their thermal hysteresis loops (The first-order transition was proved in Figure S2). Therefore, thermal hysteresis loops for each sample were calculated using the equation T_cool_ = [T_A_ + T_B_]/2, T_heat_ = [T_C_ + T_D_]/2 and T = [T_cool_ + T_heat_]/2, where A, B, C and D are the corresponding magnetic moment inflection points. From Table 2, it can be seen that T_heat_, T_cool_ (T_heat_ and T_cool_ are the phase transition temperatures in heating and cooling process, respectively) and the Tt of the material increased with Zr doping concentration increased. The T_heat_ increased slowly (0.5 K /at% doped Zr ions) with Zr doping levels increased compared with the changes of T_cool_ (4 K/at% doped Zr ions). The Zr-doped VO_2_ films prepared by Zhang et al. had the same tendency in cooling process [24]. However, Shen et al., Li et al. and Lu et al. have the opposite results with our experiment results [25,26,27]. They attributed the decreased of Tt to the changes of lattice structure. But in our experiment, except for changes of the lattice structure, the magnetic properties have a big difference after Zr doping into VO_2_ lattice, which means a mechanism associated with V-V dimers accounted for the elevated phase transition temperature.

The charge doping effect of high valence state ions normally results in materials with lower phase transition temperatures, while low valence state ions are known to increase their phase transition temperatures. This means that Zr^4+^ ions dopants do not modify the phase transition properties of VO_2_ systems through a charge doping effect. Therefore, the observed changes in the phase transition temperature are likely to be caused through a mechanism that rely on Peierls type electron phonon correlations [37,38,39]. Tomczak et al. have previously applied a lattice “Peierls substitution” method to calculate the optical conductivities of metallic and insulating states of multi-atomic unit cells and their calculated results found to be in good agreement with experimental results [37]. Kim et al. also reported that the phase transition mechanism of VO_2_ could be modelled effectively using an orbital-selective Peierls transition method that employed DFT+U calculations [38]. For VO_2_, the outermost electronic configuration of V^4+^ ion is d^1^, which means that each ion has one conducting electron. Its Fermi energy level appears at 1/2 position of the energy band, with the half empty energy states and the half occupied energy states. When V-V dimers are formed then the lattice is distorted, with the lattice constant for the quasi-1D V chains effectively doubled to produce a Fermi level with an increased energy gap that results in the decreasing of overall energy of the system. The boundary of the Brillouin zone of these distorted lattice corresponds to the Fermi level, which can change from a high energy metal state to a low energy insulated state. Then, Peierls phase transition occurred due to the fluctuation of charge density wave. Besides, S. Biermann et al. reported that VO_2_ is not a conventional Mott insulator and formation of V-V dimers plays an important role in triggering the creation of Peierls gaps in the insulted material [39]. Doping Zr into the VO_2_ system perhaps results in an overall increase in the number of V-V dimers present, with the V-V dimers of Zr requiring more energy to dissolve to enable the material to complete phase transition. We speculate that more V-V dimers are formed with Zr doping, which result in the low temperature state is more stable than high temperature state. Therefore the phase transition temperature is elevated by Zr doping in our experiment. Besides, it has previously been reported that cationic dopants with empty d orbitals are more stable in low anion coordination states, therefore Zr^4+^ is acting to stabilize the low monoclinic phase of VO_2_ [40], which results in more energy being required to complete the phase transition process. The increased number of V-V dimers has a bigger influence than lattice changes on Zr doping VO_2_ samples.

## 4. Conclusions

The un-doped, 1%, 2% and 4% Zr-doped VO_2_ powder were prepared using a hydrothermal method. It was found that Zr and V existed in the forms of Zr^4+^ and V^4+^ states, respectively. No XPS peaks corresponding to V^3+^ and V^5+^ were present in all the samples. Doping of Zr ions change the shape of the spherical un-doped VO_2_ particles into the doped VO_2_ rectangular slice shape. The saturation magnetic moments of the samples increased gradually with the increase of Zr doping concentration. The phase transition temperatures of Zr-doped VO_2_ samples were elevated from 336.07 K to 343.76 K, which were correlated to Zr ion doping concentration. We speculate that more V-V dimers are formed with Zr doping by magnetic measurements, which results in the monoclinic phase of Zr-doped VO_2_ sample is more stable than rutile phase. Therefore the phase transition temperature is elevated by Zr doping in our experiment. And the VO_2_ phase transition should be ascribed to Perils transition caused by changing of V-V dimers.

## Figures and Tables

**Table 1:** Grain size, d_011_ interplanar spacing, lattice parameters and volume of the unit cells of un-doped, 1% Zr-doped, 2% Zr-doped and 4% Zr-doped VO_2_ samples.

Sample	2θ-d_011_ (°)	d_011_ (nm)	Grain Size (nm)	a (Å)	b (Å)	c (Å)	β (°)	V = abcsin β (Å^3^)
un-doped	27.910°	3.1940	50.6	5.7435	4.5200	5.3472	122.4186	117.1826
1% Zr-doped	27.890°	3.1963	49.07	5.7604	4.5226	5.3658	122.6497	117.7008
2% Zr-doped	27.877°	3.1977	38.92	5.7578	4.5298	5.3615	122.6428	117.7497
4% Zr-doped	27.868°	3.1988	38.73	5.7607	4.5288	5.3659	122.6391	117.8845

**Table 2:** Cooled (T_cool_), heated (T_heat_) and mean phase transition temperatures (T) calculated from moment vs. temperature curves of un-doped, 1% Zr-doped, 2% Zr-doped and 4% Zr-doped VO_2_ samples.

Sample	T_cool_ (K)	T_heat_ (K)	T = [T_cool_ + T_heat_]/2 (K)
un-doped	315.06	357.08	336.07
1% Zr-doped	327.29	357.8	342.55
2% Zr-doped	327.8	358.8	343.30
4% Zr-doped	328.22	359.3	343.76

## Supplementary Materials

The following are available online at http://www.mdpi.com/2079-4991/9/1/113/s1, Figure S1: Moment vs applied field curves of un-doped VO_2_ powder samples before and after phase transition (300K and 350K), Figure S2: Specific heat capacity vs temperature of un-doped VO_2_ samples.
