# Supplementary material for: Effect of Zr Doping on the Magnetic and Phase Transition Properties of VO2 Powder
Source: Nanomaterials (Basel). 2019 Jan 18;9(1):113. doi: 10.3390/nano9010113 (PMC6359382; doi:10.3390/nano9010113)
Supplement: Supplementary file 1 [file nanomaterials-09-00113-s001.pdf]

Effect of Zr Doping on the Magnetic and Phase Transition Properties of VO_2 Powder

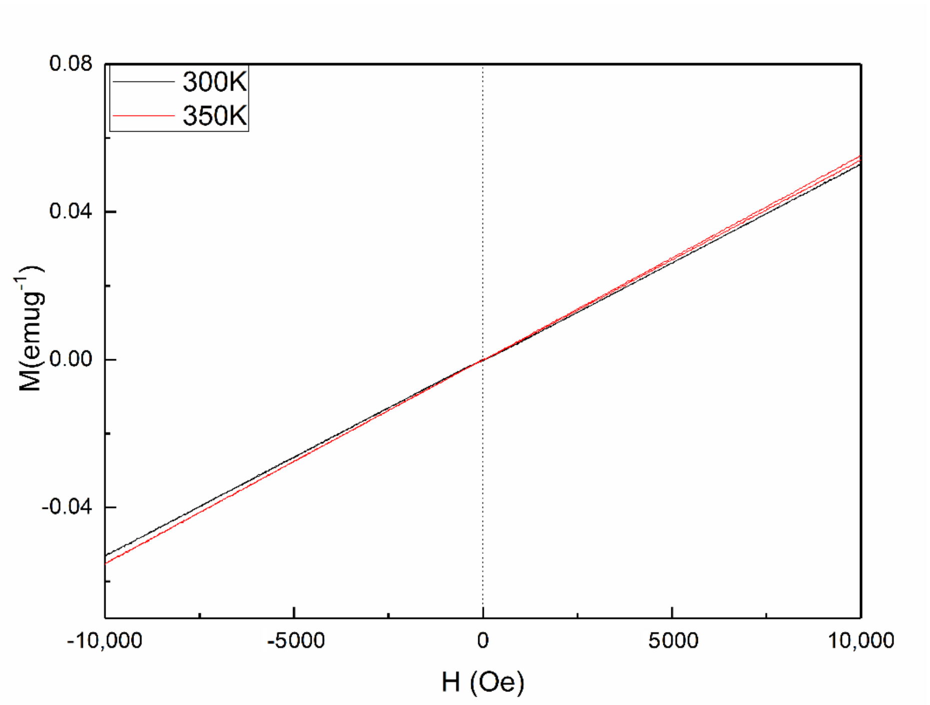

Fig. S1. Moment vs applied field curves of un-doped VO_2 powder samples before and after phase transition (300K and 350K).

From Fig.S1, the slope of un-doped VO_2 powder samples after phase transition is slightly larger than that before phase transition. But no obvious differences were observed in the samples. The un-doped VO_2 powder samples show a paramagnetic behavior before and after phase transition.

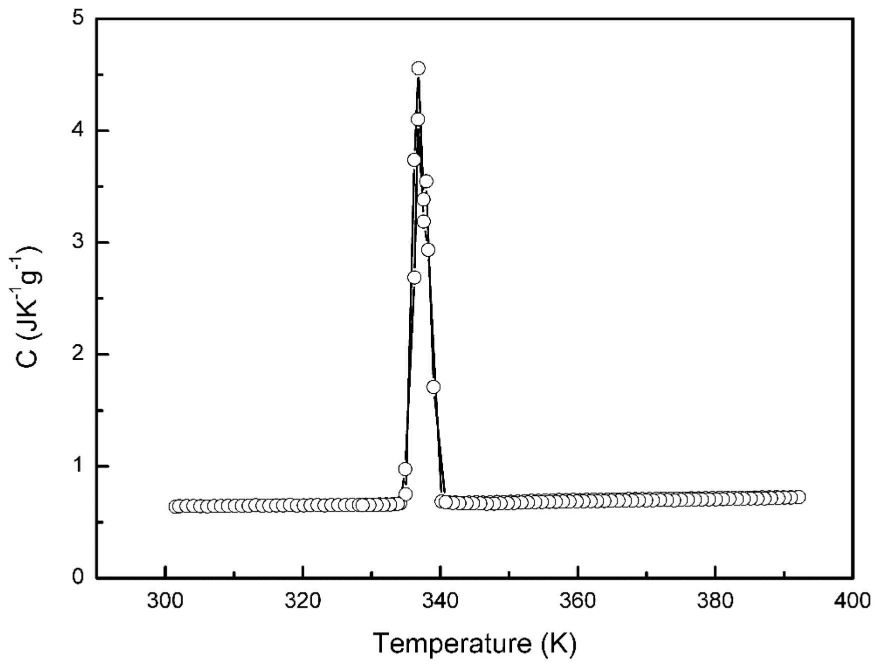

Fig. S2. Specific heat capacity vs temperature of un-doped VO_2 samples.

From Fig. S2, a sharp peak appear at phase transition temperature, which means that the phase transition of VO_2 ascribes to a first-order transition.
